# Case Report: Cytomegalovirus Disease Is an Under-Recognized Contributor to Morbidity and Mortality in Common Variable Immunodeficiency

**DOI:** 10.3389/fimmu.2022.815193

**Published:** 2022-02-15

**Authors:** Samantha Chan, Jack Godsell, Miles Horton, Anthony Farchione, Lauren J. Howson, Mai Margetts, Celina Jin, Josh Chatelier, Michelle Yong, Joseph Sasadeusz, Jo A. Douglass, Charlotte A. Slade, Vanessa L. Bryant

**Affiliations:** ^1^ Immunology Division, Walter & Eliza Hall Institute of Medical Research, Melbourne, VIC, Australia; ^2^ Department of Medical Biology, The University of Melbourne, Melbourne, VIC, Australia; ^3^ Department of Clinical Immunology & Allergy, Royal Melbourne Hospital, Melbourne, VIC, Australia; ^4^ Department of Medicine, The University of Melbourne, Melbourne, VIC, Australia; ^5^ Victorian Infectious Diseases Service, Royal Melbourne Hospital, Melbourne, VIC, Australia; ^6^ Sir Peter MacCallum Department of Oncology, The University of Melbourne, Melbourne, VIC, Australia; ^7^ National Centre for Infections in Cancer, Peter MacCallum Cancer Centre, Melbourne, VIC, Australia

**Keywords:** cytomegalovirus, herpesvirus 6, common variable immunodeficiency, predominantly antibody deficiency, primary immunodeficiencies, immunogenetics, cellular immunity

## Abstract

**Background:**

Common Variable Immunodeficiency (CVID) is classified as a ‘Predominantly Antibody Deficiency’ (PAD), but there is emerging evidence of cellular immunodeficiency in a subset of patients. This evidence includes CVID patients diagnosed with cytomegalovirus (CMV) infection, a hallmark of ‘combined immunodeficiency’. CMV infection also has the potential to drive immune dysregulation contributing to significant morbidity and mortality in CVID. We aim to determine the extent of cellular immune dysfunction in CVID patients, and whether this correlates with CMV infection status.

**Methods:**

We conducted a single-center retrospective cohort study of individuals with CVID at the Royal Melbourne Hospital, and identified patients with and without CMV disease or viraemia. We then isolated T-cells from patient and healthy donor blood samples and examined T-cell proliferation and function.

**Results:**

Six patients (7.6%, 6/79) had either CMV disease (pneumonitis or gastrointestinal disease), or symptomatic CMV viraemia. A high mortality rate in the cohort of patients with CVID and CMV disease was observed, with 4 deaths in the period of analysis (66.6%, 4/6). Individuals with CMV infection showed reduced T-cell division in response to T-cell receptor (TCR) stimulation when compared with CMV-negative patients.

**Discussion:**

This study demonstrates the morbidity and mortality associated with CMV in CVID, and highlights the need for focused interventions for patients with CVID at risk of CMV disease.

## Introduction

Predominantly Antibody Deficiency (PAD) is the most common Primary Immunodeficiency (PID) diagnosed in adults. The most prevalent PAD is Common Variable Immunodeficiency (CVID), a phenotypically heterogeneous disease characterised by hypogammaglobulinaemia, impaired vaccine responses and recurrent sinopulmonary infections (1). In addition to immunodeficiency, most individuals with CVID (>70%) also display features of chronic immune dysregulation, such as autoimmunity, malignancy and/or autoinflammation (2). These non-infectious manifestations are exceptionally challenging to manage in the context of underlying immunodeficiency.

Diagnosis of CVID typically focuses on confirming an impaired humoral response. However, emerging evidence of cellular immunodeficiency in individuals with CVID has prompted reassessment of this diagnostic framework. Reduced numbers and/or proportions of naïve CD4^+^ T-cells have been suggested as potential immunophenotypic markers for late-onset combined immunodeficiency, or, ‘LOCID’ (3–5). Viral infection is another clinically relevant hallmark of cellular immunodeficiency that has diagnostic potential in the context of CVID.

Cytomegalovirus (CMV) is a human beta-herpes virus, with high seroprevalence (40–90%) in the general adult population (6). Initial CMV infection is typically controlled, but persistent viral DNA is detectable within latent reservoirs established in undifferentiated myeloid cells (7). Viral clearance and establishment of latency occurs independent of the humoral immune response; as demonstrated by CMV infection being controlled in murine models of absolute B-cell deficiency (8). Latent CMV, once established, is also predominantly controlled by T-cell immunity (7).

CMV disease is generally defined as the presence of tissue-invasive CMV associated with an appropriate clinical syndrome (7, 9). It occurs when viral replication is reactivated, and is commonly encountered in secondary immunodeficiencies, for example, following haematopoietic stem cell transplantation (HSCT), or in the setting of acquired immunodeficiency syndrome. It was recently reported that CMV disease in CVID is relatively common and can result in fatal clinical outcomes (9). However, knowledge of CMV’s impact on the immune response in the context of CVID-associated immunodeficiency is limited.

A previous study examining T-cell responses in CVID patients with evidence of CMV exposure suggested that aberrant immune responses to CMV may directly cause inflammatory dysregulation in CVID (10). Here, an expanded population of CMV-specific late-memory T cells (CD8^+^/CD27^−^/CD28^−^) was observed, as well as an increased production of pro-inflammatory cytokines in response to CMV antigens in CVID patients with a history of CMV infection (10–12).

However, there is a lack of evidence regarding factors that precipitate CMV disease (as opposed to asymptomatic viraemia or latent infection) in CVID. Research to date mainly comprises of case reports (13–35), while larger-scale studies have included secondary immunodeficiencies (36) or focused on CMV in the setting of iatrogenic immunosuppression (37).

With the increasing use of HSCT as a curative treatment for adult PIDs, there is a pressing need to better understand the specific immune deficiencies that lead to loss of latency and development of CMV disease in the context of CVID; CMV seroprevalence is common, and active CMV disease presents a contraindication to transplant. To address this knowledge gap, we conducted a single-center study of CMV disease in CVID and explored its possible association with genetic diagnosis, T-cell proliferation and function.

## Methods

### Subject Selection

A retrospective cohort study of individuals under the care of The Royal Melbourne Hospital Clinical Immunology & Allergy Unit from 2016 to 2021 was conducted, with potential participants identified through internal auditing. The diagnosis of CVID was subsequently confirmed according to European Society for Immunodeficiencies (ESID) criteria (1). Medical records and pathology from 2016 to 2021 were interrogated for the diagnosis of CMV disease (histological evidence of tissue-invasive CMV, associated with an appropriate clinical syndrome) or symptomatic viraemia (‘CMV syndrome’), as well as factors associated with CMV infection (including lymphocyte subsets, inflammatory complications of CVID, subsequent immunosuppression, and infection history).

For functional studies, individuals with CVID and no history of clinical CMV (CVID^+^/CMV^-^) were recruited as ‘controls’ for patients with CVID and clinical CMV (CVID^+^/CMV^+^). Healthy donors (CVID^-^/CMV^-^) with no significant past medical history were enrolled through the Volunteer Blood Donor Registry (WEHI).

### Preparation of Lymphocytes and T-Cell Isolation

Peripheral blood mononuclear cells (PBMCs) were isolated from fresh whole blood by density gradient Ficoll-Leucosep centrifugation. Cells were frozen and cryopreserved in liquid nitrogen. T cells were isolated *via* negative selection using a Human T-cell Isolation Kit (Stemcell Technologies, Vancouver, Canada) following the manufacturer’s instructions. Purity of isolated CD3^+^ T-cells was >99%.

### Antibodies and Dyes

CD3-V500 (clone UCHT1) and CD4-APC (clone RPA-T4) were purchased from BD Pharmingen, San Jose, California. CD8-APC780 (clone RPA-T8), CD45RA-PeCy7 (clone HI100) and CD45RO-PE (clone UCHL1) were purchased from eBioscience, San Diego, California. CD27-FITC (clone M-T271) was purchased from Miltenyi Biotec, Bergisch Gladbach, Germany. Dead cells were excluded from analysis using propidium iodide (PI) (Sigma-Aldrich, St. Louis, Missouri). All antibody cocktails were made using Brilliant Stain Buffer (Becton Dickinson, Franklin Lakes, New Jersey).

### T-Cell Proliferation Assay

Purified T cells were labelled with CellTrace Violet (Thermo Fisher Scientific Australia, Scoresby, Australia) (38), plated in triplicate (1 x 10^4^ cells/well) and incubated for 96 hours at 37°C in the presence of: 400 U/mL IL-2 (Abcam, Boston, Massachusetts), 1 bead/cell Human T-Activator CD3/28 Dynabeads (Thermo Fisher Scientific Australia, Scoresby, Australia), or 1x PHA (Thermo Fisher Scientific Australia, Scoresby, Australia) as indicated. Data on cell counts and proportions were collected using a BD FACSCanto Clinical Flow Cytometer every 24 hours for 4 days.

Data were analyzed using FlowJo software, version 10 (Tree Star, Ashland, Oregon). The gating strategy is illustrated in **Supplementary Material (Figure X)**.

### Cytokine Assays

T cells were stimulated as described above and supernatant harvested after 48 hours, for all conditions. Quantification of IL-1β, IFN-α2, IFN-γ, TNF-α, MCP-1 (CCL2), IL-6, IL-8 (CXCL8), IL-10, IL-12p70, IL-17A, IL-18, IL-23, and IL-33 was performed using the LEGENDplex™ Human Inflammation Panel 1 (BioLegend, San Diego, California), according to the manufacturer’s instructions. Quantification of IL-2 was performed using the V-PLEX Human IL-2 kit (Meso Scale Discovery, Rockville, Maryland).

### Statistical Analysis

Statistical analyses were performed with GraphPad Prism software, version 9 (GraphPad Software, Inc, La Jolla, California). For binary outcomes, cohorts were compared using Fisher’s exact tests due to the small sample sizes. For continuous variables, Kruskal-Wallis testing was used for multiple comparisons between cohorts, with the assumption of non-parametric data distribution. Results are shown as means and error bars represent standard errors of the mean (SEM). Two-tailed P values are reported, with values of <0.05 considered statistically significant.

### Ethics

Ethical approval for the study protocol was granted by the Human Research Ethics Committees of Melbourne Health (project reference number 2009.162) and WEHI (project reference number 10/02). Written, informed consent was obtained from all participants, in accordance with the Declaration of Helsinki and subsequent amendments. For individuals who were deceased at the time of data collection, ethical approval was obtained to review their medical records.

## Results

### Clinical and Immunological Features of CVID Patients With CMV Disease

Our cohort consisted of 79 individuals with CVID. Ten patients (12.7%) had CMV Polymerase Chain Reaction (PCR) testing measured during the period of study, performed where there was clinical suspicion of CMV disease; asymptomatic CMV screening is not part of routine care at our center. Six patients (7.6%) had current or historical evidence of CMV disease or symptomatic viraemia (**Table 1** *)*. The male: female ratio was 2:1, with an age range of 31 to 60 years. Three patients were recruited for functional immunological assessment: Patient 1 (32M), Patient 2 (58F) and Patient 5 (58M).

**Table 1:** CVID^+^/CMV^+^ cohort: clinical characteristics.

PATIENT	AGE/GENDER	GENETIC DIAGNOSIS	AGE OF CVID SYMPTON ONSET	AGEAT CVID DIAGNOSIS	AGE AT CMV DIAGNOSIS (YEARS FROM CVID Dx)	INFLAMMATORY DISEASE^#^	IATROGENIC IMMUNOSUPPRESSION (YEAR ADMINISTERED)	CMV MANIFESTATION: YEAR OF DIAGNOSIS (MODE OF DIAGNOSIS)	CMV TREATMENT^	OTHER INFECTIONS	OUTCOME
1	32M	p50 haploinsufficiency (*NFKB1*); pathogenic	10	26	30 (+4)	AIHA, inflammatory arthropathy, lymphocytic enteropathy, non-cirrhotic portal hypertension	Rituximab & prednisolone (2017-2019)	Enteritis: 2018 (IHC & tissue PCR), Pneumonitis, 2021 (BAL PCR)	G, V	Chronic *Helicobacter Pylori*, pulmonary Aspergillosis, recurrent sinopulmonary infections	Recurrent disease
							Adalimumab (2021)				Deceased 2021
2	58F	p50 haploinsufficiency (*NFKB1*); pathogenic	30	38	52 (+14)	Autoimmune pancytopenia, non-cirrhotic portal hypertension: liver transplant	Everolimus, prednisolone & cyclosporin (2016-2021)	Symptomatic viraemia: 2017, 2019 (whole blood PCR)	G, V	Chronic Norovirus, recurrent *Campylobacter*, recurrent sinopulmonary infections	Treatment success, chronic viraemia
3	31M	CTLA4 haploinsufficiency; pathogenic	16	23	17 (-6)	Severe lymhocytic enteropathy, autoimmune pancytopaenia, Burkitt’s lymphoma	Hyper-CVAD (2007)	Enteritis: 2012, 2020 (IHC & tissue PCR), Pneumonitis: 2020 (BAL cytology & PCR), Chronic asymptomatic viraemia: 2016-2021 (whole blood PCR)	G, V, *V-induced neutropaenia*	Chronic Norovirus, recurrent sinopulmonary infections	Recurrent disease
							Rituximab (2007)				Deceased 2021
							Abatacept (2019-2020)				
4	60F	*TNFRSF13B* variant; risk gene	37	42	58 (+16)	Granulomatous lymphocytic interstitial lung disease – lung transplant	Rituximab & azathioprine (2017)	Enteritis: 2019 (IHC & tissue PCR)	G, V, CMVIg^+^ Lifelong suppressive V	Recurrent sinopulmonary infections	Viral suppression
							Prednisolone & tacrolimus (2017-2019)				Deceased 2019
5	58M	*ZAP70* heterozygous; variant of uncertain significance	46	50	52 (+2)	AIHA, inflammatory colitis, seronegative spondyloarthropathy	Rituximab & prednisolone (2012, 2014, 2020)	Enteritis: 2014, 2020 (IHC & tissue PCR)	G, V, CMV TCs *G-induced neutropaenia*	Oral candidiasis, recurrent Gram-negative sepsis	Recurrent disease
6	32M	IκBNS deficiency (*NFKBID*); likely pathogenic	15	19	23 (+4)	Pauci-immune crescenteric glomerulonephritis, non-cirrhotic portal hypertension, autoimmune pancytopaenia	Prednisolone (2016) Tocilizumab (2018)	Enteritis: 2018 (IHC & tissue PCR), Pneumonitis: 2018 (BAL cytology & PCR), Chronic symptomatic viraemia: 2016-2021 (whole blood PCR)	G, V	Epstein-Barr viral hepatitis, oral candidiasis	Refractory viraemia
											Deceased 2019

^#^Inflammatory disease defined as the presence of autoimmune cytopaenia, autoimmune haemolytic anaemia (AIHA), enteropathy, lymphadenopathy/splenomegaly, lymphoproliferative disease, interstitial lung disease or seronegative spondyloarthritis^^^G, ganciclovir; V, valganciclovir; CMVIg, CMV-specific immunoglobulin; CMV TCs, Adoptive CMV-specific T lymphocyte therapy; ^+^Cytogam (CSL Behring), 150 mg/kg, two infusions.

Three CVID^+^/CMV^−^ patients were selected for comparison, on the basis of age-matching (Patient 7, 35M and Patient 8, 62F), or the presence of an identical underlying monogenic defect (Patient 9, 33F, daughter of Patient 2). Clinical and immunological data on all CVID^+^/CMV^+^ and CVID^+^/CMV^−^ patients are available in **Tables 1**, **2** and **Supplementary Material (Tables 1S, 2S)**. Functional immunological analysis was undertaken on two healthy donors: a 31-year-old male and 58-year-old male (CMV serostatus not known).

**Table 2:** CVID^+^/CMV^+^ cohort: immunological characteristics.

	Patient 1	Patient 2	Patient 3	Patient 4	Patient 5	Patient 6
**Peak CMV titre,** copies/mL	2,457	18,445	1,380	113	2,382	435,757
**Total lymphocyte count,** cells x 10^9, [1.0-4.8]*	1.5	1.1	2.0	1.5	1.1	**0.1**
**CD19^+^**, cells x 10^9, [0.07-0.55]	0.07	0.11	**0.04**	0.4	0.18	**0.00**
**CD3^+^**, cells x 10^9, [0.60-2.50]	1.31	0.89	1.87	0.99	0.88	**0.07**
**CD4^+^**, cells x 10^9, [0.45-1.70]	0.5	0.51	**0.42**	0.79	0.58	**0.06**
**CD8^+^**, cells x 10^9, [0.20-1.15]	0.72	0.35	1.42	**0.16**	0.27	**0.01**
**CD4:CD8,** [1.1-2.4]	**0.69**	1.46	**0.3**	**4.94**	2.15	**6**
**CD56^+^**, cells x 10^9, [0.07-0.70]	0.12	0.1	0.08	0.11	0.07	**0.02**
**Immunoglobulin Replacement Therapy**	Intragam 10, 45g q3w (0.66 g/kg/month)	Intragam 10, 25g q2w (0.83 g/kg/month)	Intragam 10, 47.5g q42 (0.79 g/kg/month)	Intragam 10, 45g q4w (0.75 g/kg/month)	Intragam 10, 40g q4w (0.55 g/kg/month)	Intragam 10, 25g q2w (0.66 g/kg/month)
**Trough IgG at time of CMV diagnosis, g/L**	7.1 (2021)	10.0 (2019)	4.6 (2020)	8.6 (2019)	8.1 (2020)	6.9 (2018)
	6.0 (2018)	13.6 (2017)				

*[reference range]; values outside reference range in bold.

Rates of end-organ manifestations in the CVID^+^/CMV^+^ patients were as follows: CMV colitis/enterocolitis 83.3% (5/6), CMV pneumonitis 50% (3/6) and symptomatic viraemia 33.3% (2/6) add TACI mutation. A likely monogenic cause of CVID was identified in 66.7% (4/6), and the remaining 2 patients carried either a CVID risk gene (TNFRSF13B; P.Cys104arg variant) or variant of uncertain significance (ZAP70; heterozygous for c.512A>G).

All CVID^+^/CMV^+^ patients (100%, 6/6) had inflammatory manifestations of disease – for example, autoimmune cytopenias, inflammatory arthropathy, or lymphocytic colitis – with subsequent iatrogenic immunosuppression that preceded the diagnosis of CMV disease. In comparison, across the unit’s CMV-negative CVID cohort (n=73), the prevalence of inflammatory disease was 67.1% (49/73, p=0.0923) and the prevalence of immunosuppression exposure was 23.3% (17/73, p<0.0001).

Peak viral loads ranged from 113 to 435,757 copies/mL. Higher viral loads did not demonstrate clear association with severity of infection. However, it is possible that ‘true peaks’ were missed, as surveillance of CMV viral loads was not routinely performed. All patients were treated with anti-viral therapy (**Table 1**). None developed viral resistance, but two patients (Patient 3 and Patient 5) were unable to complete therapy (ganciclovir/valganciclovir) due to profound neutropaenia. Two patients required salvage therapy using adoptive CMV-specific T-cell therapy (Patient 4) and CMV-specific immunoglobulin (Patient 5). Patient 4 achieved viral suppression, but Patient 5 continues to have recurrent CMV disease.

All CVID^+^/CMV^+^ patients were on intravenous immunoglobulin replacement with physiological Immunoglobulin G (IgG) trough levels (**Table 2**), but had high rates of active bacterial infection. Each of the six patients required, on average, >2 courses of oral antibiotics annually for sinopulmonary infection, and five patients (5/6, 66.7%) were hospitalized ≥ once/year with infection over the period of study. Five of the CVID^+^/CMV^+^ cohort (5/6, 83.3%) also had a history of other opportunistic infections: chronic Norovirus, Epstein-Barr virus, chronic candidiasis and Aspergillosis. In contrast, 8.21% of the centre’s CMV-negative CVID cohort (6/73) had a history of other disseminated viral infections: varicella-zoster virus, human papillomavirus and Epstein-Barr virus.

There were 4 deaths in the CVID^+^/CMV^+^ group over the period studied. These mortalities constituted 66.7% (4/6 deaths in our cohort of 79 individuals with CVID) of unit mortalities over that time, suggesting that CMV disease increased the relative risk of death by 26.3 in individuals with CVID (Fisher’s exact test, CI 6.31-102.9; p<0.0001). Only one death was directly attributable to a complication of CMV disease (Patient 1, who died of septic shock secondary to CMV pneumonitis and pulmonary Aspergillosis). Three of the 4 patients (75%) in the CVID^+^/CMV^+^ cohort had a flare of CMV disease in the 6 months prior to death, presenting a significant barrier to immunomodulatory treatment of their inflammatory disease.

Immunophenotyping of major lymphocyte subsets (**Table 2**) revealed lymphocyte counts ≤ 2.0 x 10^9^ cells in all patients, but severe lymphopaenia only in Patient 6. One third (2/6) of patients had reduced (lower than the standard reference range for age) numbers of CD19^+^ total B cells, 16.6% (1/6) had reduced numbers of CD3^+^ T cells, 33.3% (2/6) had reduced numbers of CD4^+^ T cells, 33.3% (2/6) had reduced numbers of CD8^+^ T cells and 16.6% (1/6) had reduced numbers of CD56^+^ NK cells. The CD4:CD8 ratio was reduced in 33.3% (2/6) and elevated in 33.3% (2/6). Historical T cell immunophenotyping was available for three individuals. Naïve CD4^+^ T cells constituted 14.4% of total CD4^+^ T cells for Patient 1, 15.3% of total CD4^+^ T cells for Patient 4 and 6.74% of total CD4^+^ T cells for Patient 5, therefore only one of these patients met the Frieburg immunophenotypic criteria for combined immunodeficiency (naïve CD4^+^ T cells <10%) (5).

### Delayed Proliferative Potential of CVID+/CMV+ T Cell Populations

**Figure 1:**
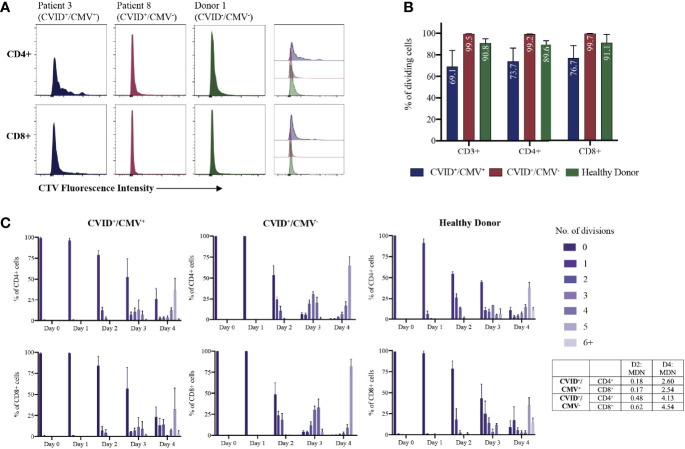
T-cell proliferation to IL-2 + CD3/28 stimulation. **(A)** Individual examples of CD4^+^ and CD8^+^ T-cell proliferation at Day 4, determined by CellTrace Violet (CTV) dilution. **(B)** Proportions of dividing cells at Day 4 for each cohort, expressed as a percentage of total CD3^+^, CD4^+^ and CD8^+^ T-cells. Results presented as means and standard errors of the mean (SEM). **(C)** T-cell proliferation over time: number of CD4^+^ and CD8^+^ divisions at each time-point, expressed as a percentage of total CD4^+^ and CD8^+^ T-cells. Results presented as means and SEM for each cohort. MDN, Mean Division Number.

We next investigated T cell proliferative responses and cytokine production in CVID^+^/CMV^+^ patients where possible (P1, P2 and P5), compared to matched CVID^+^/CMV^-^ patients (P7, P8, P9) and healthy donors. Total CD3^+^ T cells were isolated from each group, labelled with division tracking dye CTV, stimulated with IL-2, CD3/CD28, IL-2 + CD3/CD28 or PHA, harvested daily for 4 days and the proliferative potential assessed through analysis of CTV fluorescence intensity (**Figures 1A, B**).

Delayed CD3^+^ proliferation in response to IL-2 + CD3/28 stimulation was observed in the CVID^+^/CMV^+^ cohort (**Figure 1C**). On average, 78.9% of CD4^+^ T cells and 84.1% of CD8^+^ T cells in the CVID^+^/CMV^+^ group remained undivided at 48 hours, compared with 53.6% of CD4^+^ T cells and 48.5% of CD8^+^ T cells in the CVID^+^/CMV^-^ group. At the final time point (Day 4), a reduced mean proportion of T cells had undergone division (69.1%) in the CVID^+^/CMV^+^ cohort, compared to 99.5% in the CVID^+^/CMV^-^ cohort and 90.8% in healthy donors (**Figure 1B**). CVID^+^/CMV^+^ T-cells underwent, on average, fewer rounds of cell division (mean division number, MDN) than CVID patients without CMV, or healthy donors (Day 4 CD4^+^ MDN: 2.60 in CVID^+^/CMV^+^, 4.13 in CVID^+^/CMV^-^, 3.42 in healthy donors, Day 4 CD8^+^ MDN: 2.54 in CVID^+^/CMV^+^, 4.54 in CVID^+^/CMV^-^, 3.01 in healthy donors).

T-cell proliferation to PHA stimulation was diminished in both the CVID^+^/CMV^+^ and CVID^+^/CMV^-^ groups compared to healthy donors. The mean proportion of divided T cells at day 4 was 44.5% in the CVID^+^/CMV^+^ cohort, 35.3% in the CVID^+^/CMV^-^ cohort and 95.6% in healthy donors (**Supplementary Material, Figure Y**). Proportions of divided CD4^+^ cells were similar in the CVID^+^/CMV^+^ and CVID^+^/CMV^-^ groups (mean of 44.6% in CVID^+^/CMV^+^ and 44.1% in CVID^+^/CMV^-^). There was an increased proportion of divided CD8^+^ cells in the CVID^+^/CMV^+^ cohort (mean 45.5%) compared with the CVID^+^/CMV^-^ group (mean 20.4%), however results in this group were highly heterogenous (range of undivided CD8^+^ cells in the CVID^+^/CMV^+^ cohort 24.0-88.5%).

### Production of Inflammatory Cytokines by CVID+/CMV+ T Cells

**Figure 2:**
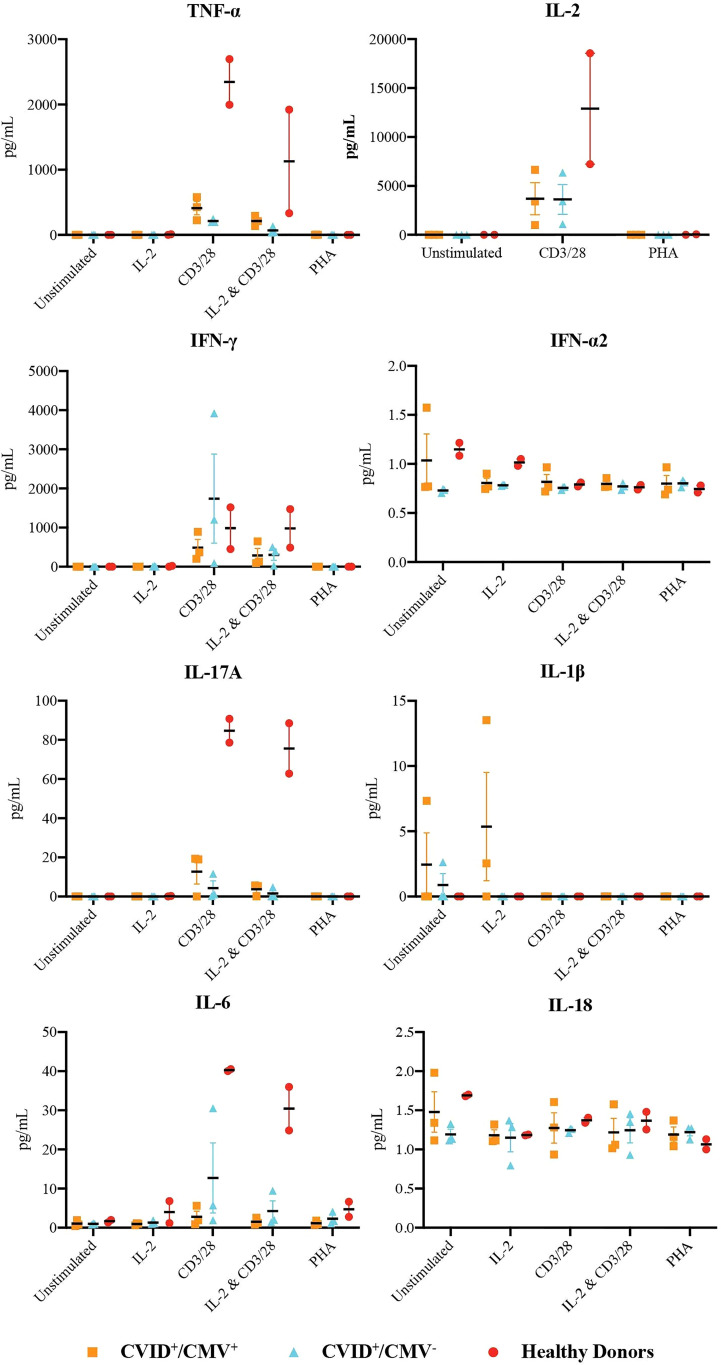
Production of inflammatory cytokines by T-cells. Concentrations of TNF-α, IL-2, IFN-γ, IFN-α2, IL-17A, IL-1b, IL-6 and IL-1 in the supernatant of proliferating T-cells at 48 hours, under all stimulation conditions. Results presented as individual values, means and standard errors of the mean.

Analysis of the supernatant from proliferating CD3^+^ T cells (**Figure 2** and **Supplementary Material, Figure Z**) largely demonstrated reduced cytokine generation in both the CVID^+^/CMV^+^ and CVID^+^/CMV^-^ cohorts in comparison to healthy donors, with the exceptions of IL-1β production in the context of IL-2 stimulation, and IFN-γ production following CD3/28 stimulation.

IFN-γ generation by CD3^+^ T-cells was highest in the CVID^+^/CMV^-^ cohort with CD3/28 stimulation (mean IFN-γ concentration 1739.1 pg/mL in the CVID^+^/CMV^-^ group, vs. 489.5 pg/mL in the CVID^+^/CMV^+^ group and 984.6 pg/mL in healthy donors), but results in this group were heavily skewed by Patient 7, who had a mean IFN-γ concentration of 3922.8 pg/mL. IL-2 + CD3/28 induced IFN-γ production was reduced in both CVID groups (289.8 pg/mL in CVID^+^/CMV^+^, 389.9 pg/mL in CVID^+^/CMV^-^) compared to healthy donors (981.1 pg/mL).

Concentrations of TNF-α, IL-2, IL-6 and IL-17A were highest in healthy donors in the presence of CD3/28 stimulation. Under these conditions, the mean concentration of TNF-α generated by CD3^+^ T cells in healthy controls was 2346.9 pg/mL (vs. 411.3 pg/mL in CVID^+^/CMV^+^ and 213.4 pg/mL in CVID^+^/CMV^-^), the mean concentration of IL-2 was 12898.9 pg/mL (vs. 3688.3 pg/mL in CVID^+^/CMV^+^ and 3623.0 pg/mL in CVID^+^/CMV^-^), the mean concentration of IL-6 was 40.26 pg/mL (vs. 2.78 pg/mL in CVID^+^/CMV^+^ and 12.73 pg/mL in CVID^+^/CMV^-^) and the mean concentration of IL-17A was 84.74 pg/mL (vs. 12.73 pg/mL in CVID^+^/CMV^+^ and 4.27 pg/mL in CVID^+^/CMV^-^).

## Discussion

The most striking clinical characteristics of our CVID^+^/CMV^+^ cohort were the high prevalence of monogenic CVID (66.7%, 4/6) and mortality rates (66.7%, 4/6 in 5 years). It is apparent from the case literature that treatment of CMV is a burdensome and often failed endeavor (9), but the potential impact of CMV disease on life expectancy in CVID has never been so starkly presented before.

Although just 7.6% of CVID patients managed by our unit had symptomatic CMV, they accounted for 66.7% (4/6) of deaths over the period of observation; therefore, the relative risk of death increased 26 times with the presence of CMV disease in this cohort. Only one death in the CVID^+^/CMV^+^ group was directly attributable to CMV. However, CMV’s broad impacts on immune health (39) and the significant barriers to its treatment (as evidenced by one-third of our patients ceasing therapy due to cytopenias) suggest that CMV’s indirect effects on morbidity and mortality in PID may be more significant than previously recognized.

Given that monogenic causes of disease are generally identified in less than 20% of CVID (40), our finding of pathogenic variants in 4 of 6 patients is noteworthy. The frequency of mutations relating to the *NFKB* pathway in this group (3/6, 50%) is also of interest, given a recent report on overwhelming CMV infection in the context of a novel *NFKB2* mutation with reduced NK cell function (14). As the evidence base grows, it may be possible to propose further specific genetic abnormalities that contribute to CMV risk.

To our knowledge, this is the largest published case series of CMV disease in CVID. However, our finding of 6 cases of CMV disease in 79 individuals with CVID is comparable to the previously published finding of 3 affected patients in 32 individuals with CVID followed up for 335 patient-years (41).

‘True’ rates of CMV exposure, viraemia and disease are exceptionally difficult to evaluate in the context of PAD. Measurement of CMV-specific IgG is misleading in the setting of immunoglobulin replacement, use of serial molecular testing (CMV PCR) is a poorly established and costly assay, and tissue biopsy may carry high ‘false negative’ rates. For instance: the presence of CMV-specific CD8^+^ T cells was reported in 55% of a UK CVID cohort (n=76), but genomic viral DNA was not detected in whole blood PCR (sensitivity 200 copies/mL of blood) in any of these patients, nor were CMV inclusion bodies demonstrated in three individuals strongly suspected to have CMV enteritis (11). On the other hand, another UK-based study found persistent peripheral CMV gene fragments in 4.9% (5/102) of their CVID patients, without any evidence of clinically relevant CMV disease in that cohort (42).

Rates of inflammatory disease in our patients with CMV disease (100%) were higher than the frequency reported in the pooled literature (77%) (9). This could be considered support for the hypothesis that an unrestrained T-cell response to CMV drives end-organ inflammation in CVID, even in the absence of detectable CMV viraemia or diagnostic histology. In a cohort of 42 patients with CVID, 73.8% (31/42) of individuals with inflammatory manifestations of CVID (hepatitis, splenomegaly, enteropathy or interstitial lung disease directly attributed to CVID) showed evidence of CMV exposure, vs. 25.8% (8/31) of individuals with an ‘infections-only’ phenotype (10).

It should be noted that lack of standardization in CMV testing is likely to result in a degree of ascertainment bias. Clinicians’ threshold for CMV investigation is presumed to be lower where there is a history of significant iatrogenic immunosuppression, presenting a significant confounder to the apparent association between CMV and inflammatory disease. An alternative hypothesis to that of CMV directly causing inflammatory manifestations of CVID, is that individuals with inflammatory CVID are more likely to undergo genetic testing, more likely to require immunomodulatory treatment, and more likely to be investigated for CMV disease.

Our studies of T-cell proliferation and function were limited by the confounding factor of iatrogenic immunosuppression, small sample sizes, and lack of longitudinal data; in particular, samples pre-and post-onset of CMV disease would be valuable. Additionally, we were unable to analyse isolated CD4^+^/CD8^+^ T-cell proliferation or perform more extensive T-cell immunophenotyping for evaluation of LOCID, due to the low numbers of cells available for processing (in many cases due to the significant mortality rates in this cohort).

Most likely as a result of the small sample size, differences in the proportions of undivided to divided cells across the CVID^+^/CMV^+^, CVID^+^/CMV^-^ and healthy donor groups did not reach statistical significance for any of the time points and conditions studied. Similarly, there were no statistically significant differences between the three groups regarding cytokine concentrations in the supernatant produced by proliferating CD3^+^ T cells at 48 hours. Nonetheless, several interesting trends emerged.

The finding of delayed CD3^+^ proliferation to both CD3/28 activator and PHA has been demonstrated in previous case studies (23, 29), but is inconsistent with a report showing increased Ki-67 expression and decreased PD-1 expression (suggesting increased cell turnover and reduced cell exhaustion respectively) by CD8^+^ CMV-specific T cells in patients with CVID previously exposed to CMV (10). Perhaps the functional profile of CD8^+^ CMV-specific T cells is distinct from that of the wider CD3^+^ T cell population. This may carry implications for inflammation, but is arguably less relevant to opportunistic infection risk than the broader assays undertaken in this study.

Kuntz et al.’s analysis of bulk CD8^+^ T cells in 34 CVID patients demonstrated reduced numbers of CCR7^+^ CD8^+^ T cells and PD-1^+^ CD8^+^ T cells in individuals with CVID previously exposed to CMV, suggesting a more differentiated immunophenotype (29). This is in keeping with previous speculation that chronic CMV infection drives ‘immuno-senescence’, chiefly through clonal expansion of dysfunctional CD8^+^ CD28^-^ T cells that are anergic to stimulation with specific antigen (43).

Our analysis of supernatant cytokine concentrations is in contrast with previous studies reporting similar or higher levels of IFN-γ and TNF-α production in individuals with CVID compared with healthy donors. However, these experiments have typically been performed on virus-specific CD8^+^ T cells (10). Furthermore, the spread of results in our supernatant experiments, particularly within the CVID^+^/CMV^-^ group, limit the reliability of this measure.

This single-centre retrospective cohort study suggests that CMV disease is a under-recognised manifestation of CVID and under-appreciated contributor to morbidity and mortality, particularly in the context of inflammatory disease and immunosuppression. An evidence base for CMV screening and treatment is sorely needed, given the significant challenges of treating CMV in the context of CVID, and CMV’s potential implications for the possibility of curative treatment with HSCT. Assessment of CMV-specific T cell immunity, such as measurement of IFN-γ release by T cells in response to to CMV antigen (using either ELISPOT or QuantiFERON), are promising new assays for investigation of CMV-specific T cell deficits. However, these tests are not yet validated in PID (44–47).

Research in this area is hampered by the rarity and heterogeneity of PID. A targeted study of the CVID population presents an appealing launching pad for research to improve risk stratification, early identification and treatment of CMV disease: a significant knowledge gap in contemporary PAD management, given the potential sequelae of CMV disease described in the literature and reiterated in our cohort study.

## Data Availability Statement

The original contributions presented in the study are included in the article/**Supplementary Material**. Further inquiries can be directed to the corresponding author.

## Ethics Statement

The studies involving human participants were reviewed and approved by the Human Research Ethics Committees of Melbourne Health (project reference number 2009.162) and WEHI (project reference number 10/02). The patients/participants provided their written informed consent to participate in this study.

## Author Contributions

SC and JG wrote the manuscript. CS and VB conceptualised and designed the study and supervised the project. SC, MH, LH, AF, and MM performed the experiments and analysed the data. SC, JG, CJ, JC, JS, MY, JD, and CS provided the clinical datasets. All authors contributed to the article and approved the submitted version.

## Funding

The authors acknowledge the Melbourne Genomics Health Alliance, supported by the Victorian Government and Alliance members, and the Australian National Health and Medical Research Council (NHMRC, Project Grant 1127198 for VB). SC receives support through a WEHI Scientific Excellence PhD Scholarship. VB and CS are supported by Sir Clive McPherson Family Research Fellowships. VB is also supported by the Royal Melbourne Hospital DW Keir Fellowship, the Victorian State Government Operational Infrastructure Scheme and Australian Government NHMRC IRIISS, the Pam and Harold Holmes Foundation, and a WEHI Innovation Grant. CS also receives support from the Scobie and Claire Mackinnon Trust. The funders were not involved in the study design, collection, analysis, interpretation of data, the writing of this article or the decision to submit it for publication.

## Conflict of Interest

SC reports grants, personal fees and nonfinancial support from CSL and nonfinancial support from Sanofi outside the submitted work. She has undertaken contracted research on behalf of: Grifols, CSL, BioCryst & Equilium. JS has received research funding from Gilead Sciences and the NHMRC. MY has received honoraria from MSD. In the past 5 years, JD has received honoraria for educational presentations from Astra-Zeneca, GSK, Novartis & CSL. She has served on advisory boards for Sanofi-Aventis, Novartis, GSK, Astra-Zeneca, Immunosis and CSL. She has undertaken contracted or investigator initiated research on behalf of: GSK, Novartis, Immunosis, AstraZeneca, Sanofi-Aventis, Grifols, CSL, BioCryst & Equilium. She has a personal superannuation shareholding in CSL and received book royalties from ‘Fast Facts: Asthma’. CS has served as a medical advisor to Grifols, Takeda and CSL and has undertaken contracted or investigator initiated research on behalf of: Takeda, Grifols, CSL & Immunosis. VB has undertaken investigator initiated research on behalf of Immunosis.

The remaining authors declare that the research was conducted in the absence of any commercial or financial relationships that could be construed as a potential conflict of interest.

## Publisher’s Note

All claims expressed in this article are solely those of the authors and do not necessarily represent those of their affiliated organizations, or those of the publisher, the editors and the reviewers. Any product that may be evaluated in this article, or claim that may be made by its manufacturer, is not guaranteed or endorsed by the publisher.
