# Supplementary material for: Case Report: Cytomegalovirus Disease Is an Under-Recognized Contributor to Morbidity and Mortality in Common Variable Immunodeficiency
Source: Front Immunol. 2022 Feb 15;13:815193. doi: 10.3389/fimmu.2022.815193 (PMC8885594; doi:10.3389/fimmu.2022.815193)
Supplement: Supplementary file 1 [file DataSheet_1.pdf]

Table 1S. $CVID^{+}/CMV^{-}$ cohort: clinical characteristics.

PATIENT	AGE/ GENDER	GENETIC DIAGNOSIS	AGE OF CVID SYMPTON ONSET	AGEAT CVID DIAGNOSIS/	INFLAMMATORY DISEASE#	IATROGENIC IMMUNOSUPPRESSION	INFECTIONS
7	35M	No pathogenic variant identified	25	34	Nil	Nil	Recurrent sinopulmonary & soft tissue infections
8	62F	No pathogenic variant identified	31	45	Lymphadenopathy	Nil	Recurrent sinopulmonary infections, small intestinal bacterial overgrowth
9	33F	*NFKB1*: c.730+4A>G	16	29	Lymphocytic enteropathy, mild bronchiectasis	Nil	Recurrent sinopulmonary infections

Inflammatory disease defined as the presence of autoimmune cytopaenias, autoimmune haemolytic anaemia (AIHA), enteropathy, lymphadenopathy/splenomegaly, lymphoproliferative disease, interstitial lung disease or seronegative spondyloarthritis, WES: Whole Exome Sequencing.

Table 2S. CVID^{+}/CMV^{-} cohort: immunological characteristics.

	**Patient 7**	**Patient 8**	**Patient 9**
**Total lymphocyte count,** cells x 10^9, [1.0-4.8]*	1.8	6.7	3.2
**CD19+**, cells x 10^9, [0.07-0.55]	**0.02**	1.52	0.72
**CD3+**, cells x 10^9, [0.60-2.50]	1.64	**4.93**	2.07
**CD4+**, cells x 10^9, [0.45-1.70]	1.02	3.88	1.41
**CD8+**, cells x 10^9, [0.20-1.15]	0.59	0.91	0.56
**CD4:CD8,** [1.1-2.4]	1.74:1	**4.27:1**	2.49:1
**CD56+**, cells x 10^9, [0.07-0.70]	0.16	0.19	0.32

*[reference range]; values outside reference range in **bold.**
