# Supplementary figures and images for: Case Report: Cytomegalovirus Disease Is an Under-Recognized Contributor to Morbidity and Mortality in Common Variable Immunodeficiency
Source: Front Immunol. 2022 Feb 15;13:815193. doi: 10.3389/fimmu.2022.815193 (PMC8885594; doi:10.3389/fimmu.2022.815193)

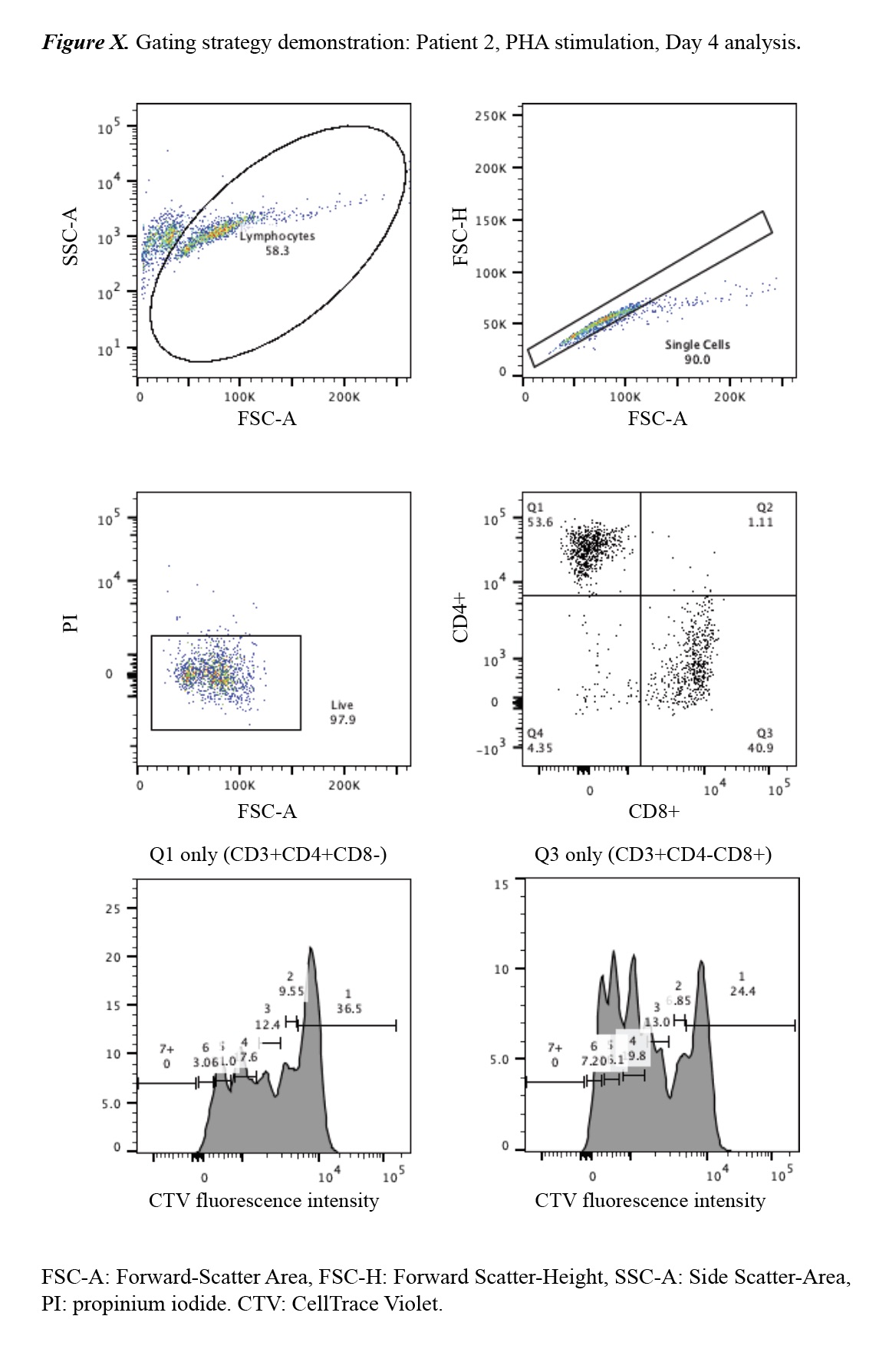

Supplement: Supplementary file 2 [file Image_1.jpeg]

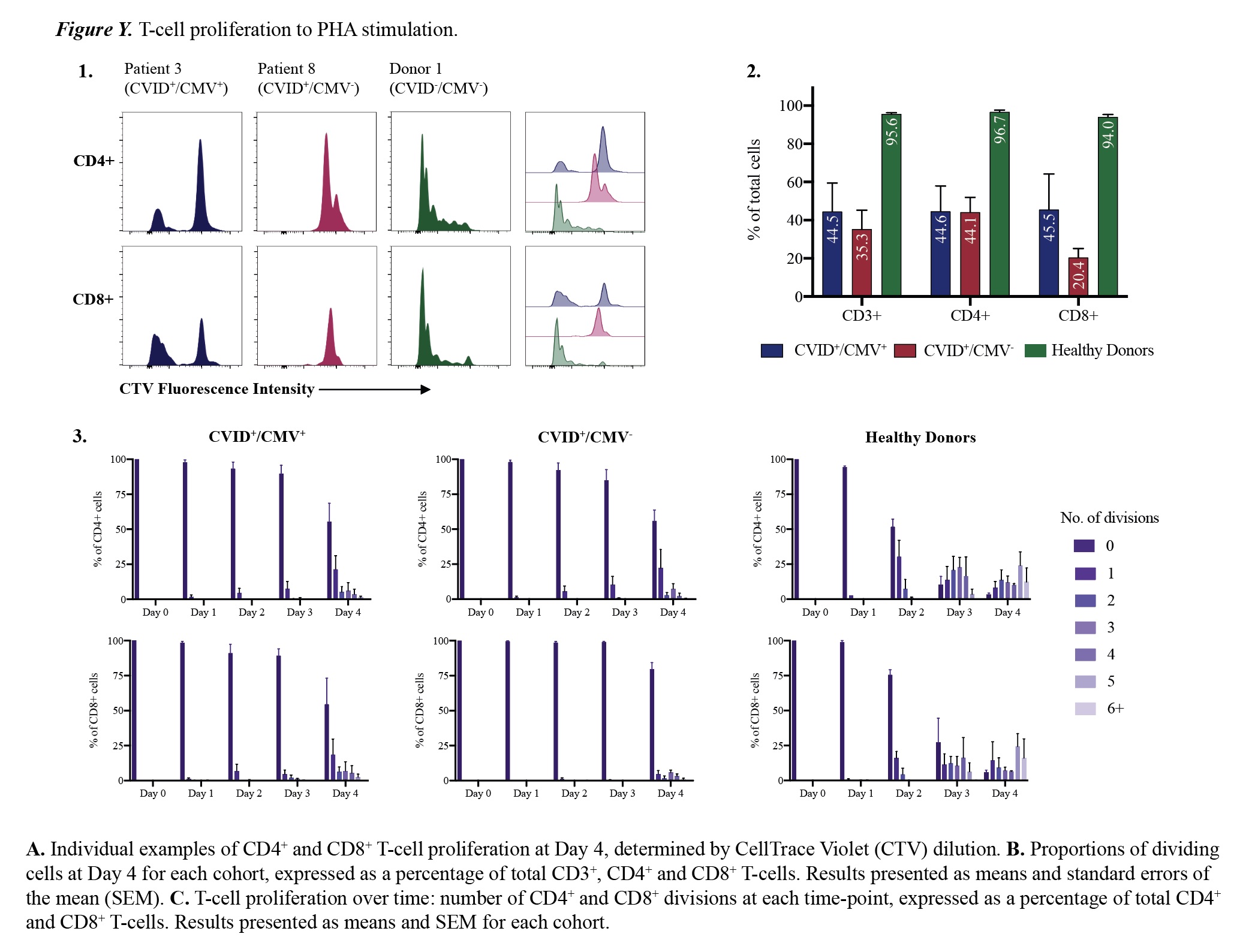

Supplement: Supplementary file 3 [file Image_2.jpeg]

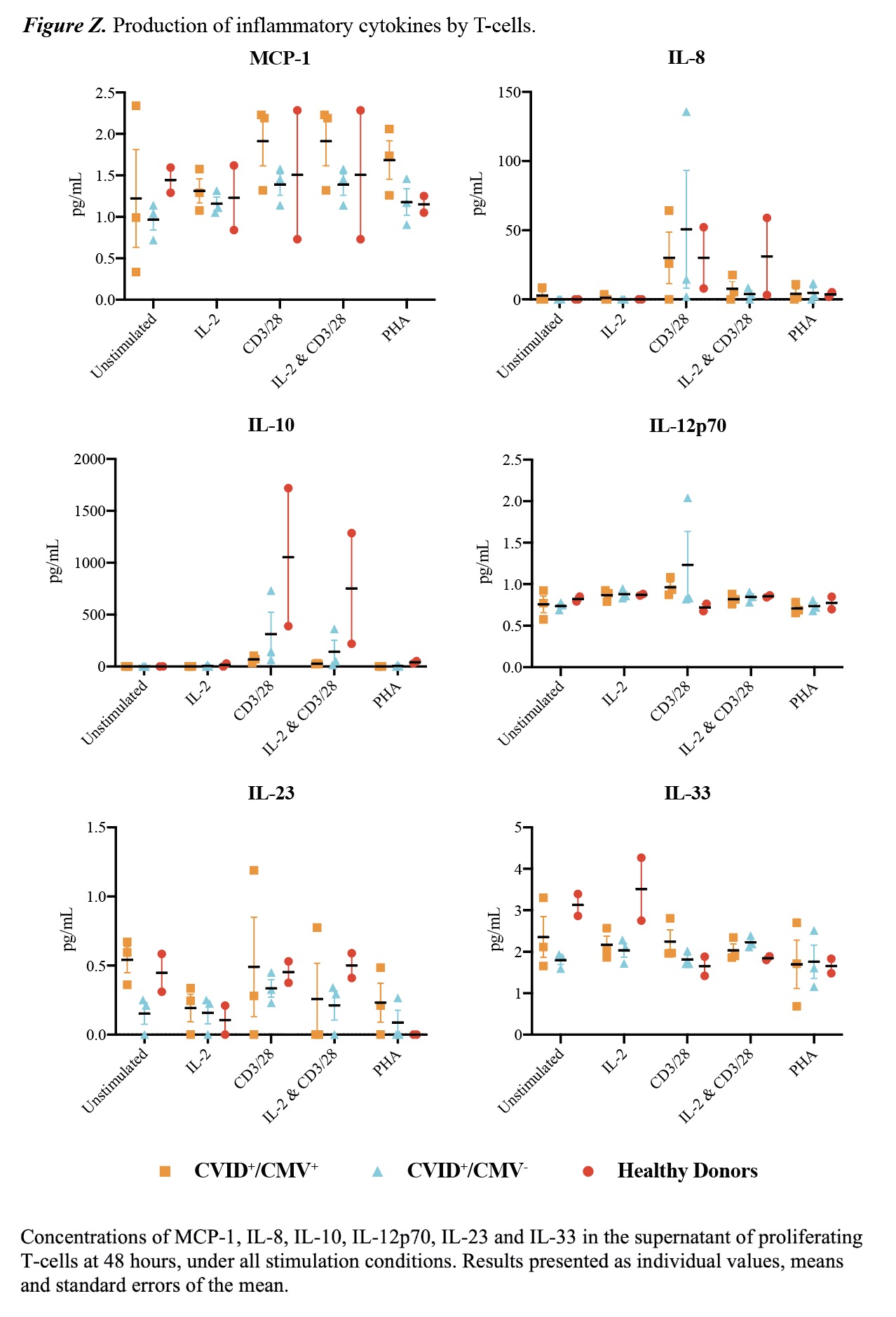

Supplement: Supplementary file 4 [file Image_3.jpeg]
